# 

**Published:** 1839-01-01

**Authors:** 


### CONTENTS

OF THE

### MEDICO-CHIRURGICAL REVIEW,

### No. LIX. JANUARY 1, 1839,

### [BEING No. XIX. OF A DECENNIAL SERIES.]

### REVIEWS.

I.

1. Urinary Diseases and their Treatment. By ROBERT WILLIS, M.D. .. .. ..
2. Traité des Maladies des Reins, étudiées en elles-memes et dans leurs Rapports avec les Maladies des Ureteres de la Vessie, de la Prostate, de l'Urèthre. Par P. RAYER, M.D. .. .. .. .. .. .. .. .. .. .. .. .. 1

1. Functional Derangements of the Kidneys and their immediate Consequences .. 6
   A. Morbid States in which the Secreting Faculty of the Kidneys is exalted .. 6
   B. Morbid States in which the Secreting Faculty of the Kidneys is lessened or abolished .. .. .. .. .. .. .. .. .. .. .. .. .. .. .. 14
   C. Morbid States in which the Urine contains in excess Ingredients that occur normally in smaller quantity .. .. .. .. .. .. .. .. .. .. .. 18

II.

Des Maladies Mentales. Par E. ESQUIROL .. .. .. .. .. .. .. .. .. .. .. 37

III.

Statistical Report on the Sickness, Mortality, and Invaliding, among the Troops in the West Indies .. .. .. .. .. .. .. .. .. .. .. .. .. .. .. .. .. 49

IV.

A Treatise on Neuralgia. By RICHARD ROWLAND, M.D. .. .. .. .. .. .. .. 63
1. Exciting Causes .. .. .. .. .. .. .. .. .. .. .. .. .. .. .. .. .. 64
2. Seat of Neuralgia .. .. .. .. .. .. .. .. .. .. .. .. .. .. .. .. .. 65
3. Pathology .. .. .. .. .. .. .. .. .. .. .. .. .. .. .. .. .. .. 66
4. Treatment .. .. .. .. .. .. .. .. .. .. .. .. .. .. .. .. .. .. 66

V.

An Experimental Essay on the Physiology of the Blood. By CHARLES MAITLAND, M. D. .. .. .. .. .. .. .. .. .. .. .. .. .. .. .. .. .. .. 69

VI.

Transactions of the Medical and Physical Society of Bombay, Vol. I. .. .. .. .. .. 73
1. Cases of Cardiac Disease and of Tubercular Phthisis occurring in the 2nd Regiment .. .. .. .. .. .. .. .. .. .. .. .. .. .. .. .. .. 74

VII.

Medico-Chirurgical Transactions, Vol. XXI. .. .. .. .. .. .. .. .. .. .. 76
1. Note on the comparative prevalence of Calculous Diseases, &c. By Mr. COPLAND HUTCHISON .. .. .. .. .. .. .. .. .. .. .. .. .. .. 77
2. History of a Female who has four Mammæ and Nipples By ROBERT LEE, M.D. 78
3. Results of Poisoning by Sulphuric Acid. By JOHN WILSON, M.D... .. .. .. 79
4. History of a Case of Popliteal Aneurysm. By SAMUEL HADWEN, Esq. .. .. 80
5. Account of a Case of Enormous Ventral Aneurysm, with the Post-mortem Appearances. By Sir DAVID J. H. DICKSON, M.D. .. .. .. .. .. .. .. 84
6. On Necrosis; being an Experimental Inquiry into the Agency ascribed to the Absorbents in the Removal of the Sequestrum. By GEORGE GULLIVER, Esq. 86
7. On the proportions of Animal and Earthy Matter in the different Bones of the Human Body. By G. O. REES, M.D. .. .. .. .. .. .. .. .. .. .. 90
8. Facts and Inferences relative to the Condition of the Vital Organs and Viscera in general, as to their Condition in certain Chronic Diseases. By JOHN CLENDINNING, M.D. .. .. .. .. .. .. .. .. .. .. .. .. .. .. .. 93

9. On Increased Thickness of the Parietes of one of the Ventricles of the Heart, with Diminution of its Cavity. By G. Budd, M.B. .. .. .. .. .. .. 98
10. On Aneurysm of the Heart, with Cases. By John Thurnam, Esq. .. .. 102
A. Aneurysm of the Ventricles .. .. .. .. .. .. .. .. .. .. .. .. 102
B. Aneurysm of the Auricles .. .. .. .. .. .. .. .. .. .. .. .. 109
C. Aneurysm of the Valves of the Heart .. .. .. .. .. .. .. .. .. .. 110

VIII.

Report of the Malignant Fever called the "Pali Plague," which has prevailed in some parts of Rajpootana, since July 1836. By James Rankin, M.D. .. .. 112

IX.

An Experimental Essay on the Relative Physiological and Medicinal Properties of Iodine and its Compounds. By Charles Cogswell, M.D. .. .. .. .. 116

X.

A Treatise on Inflammation. By James Macartney, M.D. .. .. .. .. .. .. 119
A. Consequences of Inflammation .. .. .. .. .. .. .. .. .. .. .. 123
B. On the different Modes of Reparation .. .. .. .. .. .. .. .. .. 128
C. Constitutional or remote Causes of Inflammation .. .. .. .. .. .. 132
D. On the Remedies for Inflammation .. .. .. .. .. .. .. .. .. .. 136

XI.

Physiological Explanation of the Beauty of Form. By Benjamin F. Joslyn, M.D. .. 143

XII.

Third Fasciculus of Anatomical Drawings, selected from the Collection of Morbid Anatomy in the Army Medical Museum at Chatham. Drawn by Waterhouse Hawkins .. .. .. .. .. .. .. .. .. .. .. .. .. .. 146

XIII.

Practical Remarks on the Diseases of the Skin, on the External Signs of Disorder, and on the Constitutional Peculiarities during Infancy and Childhood. By Walter C. Dendy, Esq. .. .. .. .. .. .. .. .. .. .. .. .. .. 152

### PERISCOPE.

### Bibliographical Notices.

1. On the Successful Treatment of Consumptive Disorders, and Female Complaints connected therewith; on Scrofulous Diseases, &c. By J. J. Furnival, M.D. .. 157
2. On the Improvement of Medicine. By Theophilus Thompson, M.D. .. .. .. 160
3. On the Causes of Epidemic Fever in the Metropolis, especially among the labouring classes. By Holt Yates, M.D. .. .. .. .. .. .. .. .. .. .. .. 162
4. Observations on the Oriental Plague, and on Quarantines, &c. By John Bowring, Esq. .. .. .. .. .. .. .. .. .. .. .. .. .. .. .. 163
5. The Medical Annual or British Medical Almanack, 1839. Edited by William Farr 164
1. On Hospital Medical Staffs .. .. .. .. .. .. .. .. .. .. .. .. 164
2. The Barber-Surgeons and Surgeons not Barbers.. .. .. .. .. .. .. 165
3. Temperance Societies in America.. .. .. .. .. .. .. .. .. .. .. 166
6. An Account of some New Instruments for Tying Polypi of the Uterus, Nose and Ear, and Enlarged Tonsils; with Cases. By William Beaumont, Esq. .. .. 167

RECENT WORKS ON SURGERY.

7. A System of Practical Surgery, with numerous Explanatory Plates. By John Lizars, Esq. .. .. .. .. .. .. .. .. .. .. .. .. .. .. .. 168
8. The Principles of Surgery, Vols. I. and II. By John Burns, M.D. .. .. .. 168
1. Lancet Punctures in Inflammation .. .. .. .. .. .. .. .. .. .. 169
2. Chronic Hypertrophy of the Scalp .. .. .. .. .. .. .. .. .. .. 170
3. Linear Admeasurements for finding and securing the Femoral Artery .. .. 170
4. Diseases of the Bursæ and Bunions .. .. .. .. .. .. .. .. .. .. 170
5. Malformations of the Shoulder and Hip Joints .. .. .. .. .. .. .. 171

RECENT WORKS ON ANATOMY.

9. Manual of Descriptive and Pathological Anatomy. By J. F. Meckel .. .. .. 173
10. Practical and Surgical Anatomy. By J. E. Wilson, Esq. .. .. .. .. .. 174

11. A Text Book of Human Anatomy, designed to facilitate the Study of that Science. By Robert Hunter, M.D. .. .. .. .. .. .. .. .. . .. .. .. .. 174

12. The Science of the Cerebro-spinal Phenomena attempted. By John S. Waugh, M.D. 174

13. Outlines of Human Osteology. By F. O. Warde, Esq. .. .. .. .. .. .. .. 174

14. The Surgical Anatomy of the Perinæum. By Thomas Morton .. .. .. .. 174

15. Quain's Plates. Fasciculi 62 and 63 .. .. .. .. .. .. .. .. .. .. 174

NATURAL HISTORY.

16. A History of British Birds. By William Yarrell, F.L.S. Part IX... .. .. .. 176
17. A General Outline of the Animal Kingdom. By Thomas Rymer Jones, F.Z.S. .. 176

### Clinical Review, and Hospital Reports.

GUY'S HOSPITAL.

1. Guy's Hospital Reports, No. 7. .. .. .. .. .. .. . .. .. .. .. .. 177
1. A Contribution to the Pathology of Congenital Deafness. By Edward Cock.. 177
2. On the Effect produced upon the Pulse by a Change of Posture. By William A. Guy, M.B. .. .. .. .. .. .. .. .. .. .. .. .. .. .. .. 182
A. Influence of Sex .. .. .. .. .. .. .. .. .. .. .. .. .. .. 182
B. Influence of the Period of the Day .. .. .. .. .. .. .. .. .. 183
C. Experiments with the Horizontal Board .. .. .. .. .. .. .. 183
3. Observations on Intus-susception as it occurs in Infants .. .. .. .. .. 184
A. Inflammatory Intus-susception .. .. .. .. .. .. .. .. .. .. 185
4. Chemical Examination of the Liquor Amnii. By G. O. Rees, M.D.. .. .. 188
5. On Diabetic Blood. By G. O. Rees, M.D. .. .. .. .. .. .. .. .. 190
6. Some Observations on the Cause of Strangulation in Hernia. By Mr. T. King 191
7. Cases and Observations on Medical Jurisprudence. By Alfred S. Taylor, Esq. 193
A. Survival after extensive Rupture of the Diaphragm .. .. .. .. .. 193
B. Open Foramen Ovale .. .. .. .. .. .. .. .. .. .. .. .. .. 194
8. Observations on Abdominal Tumors and Intumescence. By R. Bright, M.D... 194
A. Alterations of the Substance of the Spleen.. .. .. .. .. .. .. 194
B. Ulcerations of the Tunics of the Spleen .. .. .. .. .. .. .. .. 197
C. Symptoms .. .. .. .. .. .. .. .. .. .. .. .. .. .. .. 198
9. Physiological Observations on the Muscles of the Eye. By Bransby B. Cooper, F.R.S. .. .. .. .. .. .. .. .. .. .. .. .. .. .. .. .. 201
2. Hæmatology—M. Magendie's Lectures on the Blood.. .. .. .. .. .. .. 203
3. An Introductory Discourse on the Studies required for the Medical Profession. By Sir Benjamin C. Brodie, Bart... .. .. .. .. .. .. .. .. .. .. .. 211
4. Clinical Observations on the Primary Treatment of Injuries, delivered at the New York Hospital. By A. H. Stevens, M.D. .. .. .. .. .. .. .. .. .. 216

NEW YORK HOSPITAL.

5. Lectures on Lithotomy delivered at the New York Hospital. By A. H. Stevens, M.D... .. .. .. .. .. .. .. .. .. .. .. . .. .. .. .. 219
A. On the Causes of Death from Lithotomy .. .. .. .. .. .. .. 219

ROYAL INFIRMARY OF EDINBURGH.

6. The Dangers of Lithotrity. By W. Fergusson, F.R.C.S.E. .. .. .. .. .. 223

### Spirit of the Foreign Periodicals, &c.

1. Discussion at the Royal Academy on the Contagiousness of Plague and other Fevers 225
2. Discussion at the Royal Academy of Medicine on the Etiology of Club Feet .. 227
3. Physiological and Therapeutical Examination of Veratria. By Dr. Forche .. .. 228
4. Incontinence of Urine successfully treated with the Nux Vomica .. .. .. .. 230
5. On the Use of Chlorine Inhalation in Catarrh .. .. .. .. .. .. .. .. 231
6. Remarks on, with case of, Pneumo-Thorax .. .. .. .. .. .. .. .. 232
7. Observations on the Perforation of the Intestine by Worms. By Dr. Monchiere.. 233
8. Expulsion of a Long Loop of Intestinal Canal by the Rectum .. .. .. .. 237
9. Case of Remarkable Pulsation in all the Superficial Veins of the Arms and Neck.. 237
10. The 'Soupe à la Minute,' recommended by Baron Percy .. .. .. .. .. .. 239
11. Remarks on some curious cases of Simulation .. .. .. .. .. .. .. .. 240
12. On the Presence of Urea in the Blood.. .. .. .. .. .. .. .. .. .. 241
13. On the Action of Diuretic Medicines .. .. .. .. .. .. .. .. .. .. 243
14. Efficacy of Mercury as an Antiphlogistic Remedy .. .. .. .. .. .. .. 245
A. Mercurial Inunction in Peritonitis.. .. .. .. .. .. .. .. .. .. 247

15. Inflammation of the Umbilical Vein in Infants.. .. .. .. .. .. .. .. .. .. .. 247
16. On Foundling and Bastard Children in France and in other Countries of Europe.. 248
17. On the danger of being Buried Alive, and of the Means to ascertain Death .. .. 250
18. Poisoning by Arsenic—Exhumation of the Body, and Conviction of the Criminal three years after Death .. .. .. .. .. .. .. .. .. .. .. .. .. .. .. 251
19. Case of Torticollis, or Wry-Neck, treated by Division of the Sterno-Mastoid Muscle 253
20. Fistula of the Corpus Spongiosum Urethræ treated by Suture.. .. .. .. .. .. 255
21. Case of Hydatidic Goitre, or Hydrocele of the Neck, successfully treated by Operation .. .. .. .. .. .. .. .. .. .. .. .. .. .. .. .. .. .. 256
22. Lipoma of large size in the Groin successfully extirpated.. .. .. .. .. .. .. 257
23. Surgical Report of the Palermo Hospital .. .. .. .. .. .. .. .. .. .. .. 258
A. Case of Fungus Hæmatodes .. .. .. .. .. .. .. .. .. .. .. .. .. .. 258
B. Urinary Calculi in the Female mistaken for Diseased Uterus .. .. .. 258
24. On the Application of Raw Cotton to Erysipelatous Surfaces.. .. .. .. .. .. 260
25. The Pellicle of an Egg, an excellent Adhesive Application to Wounds .. .. .. 261
26. Diffuse Inflammation of the Scalp, in which a large extent of the Cranium exfoliated, and the patient recovered .. .. .. .. .. .. .. .. .. .. .. .. 261
27. Encysted Tumors—Serous, Atheromatous and Meliceric—treated by Incision and Injections .. .. .. .. .. .. .. .. .. .. .. .. .. .. .. .. .. .. 262
28. Case of Ileus in which Gastrotomy was performed—Death .. .. .. .. .. .. 264
29. Case of Chronic Abscess in the Left Iliac Fossa, bursting into Intestine—Cure .. 265
30. On the most frequent Causes of Sudden Death. By M. Devergie .. .. .. .. .. 266
31. Delirium Tremens, History, and Treatment of, with Emetics. By Stoll .. .. 269
32. On the State of Vaccination in various Countries of Europe; propriety of Re-Vaccination, &c. .. .. .. .. .. .. .. .. .. .. .. .. .. .. .. .. 271

### Miscellanies.

1. Researches on Suppuration. By George Gulliver, Esq. .. .. .. .. .. .. .. 273
2. On the Present State of the Laws of Lunacy. By a Barrister .. .. .. .. .. 277
3. Law of Lunacy. The Monthly Chronicle.. .. .. .. .. .. .. .. .. .. 277
4. Hæmatophobia, or the Beauties of Continental Practice.. .. .. .. .. .. .. 280
5. Saratoga Springs .. .. .. .. .. .. .. .. .. .. .. .. .. .. .. .. .. 281
6. Medical Reform. By A. B. Granville, M.D. .. .. .. .. .. .. .. .. .. .. 282
7. The late Mr. Lockley .. .. .. .. .. .. .. .. .. .. .. .. .. .. .. .. 284
8. Dialogue between a Physician and a Physiologist .. .. .. .. .. .. .. .. 287
9. Dr. Granville and Dr. Roe .. .. .. .. .. .. .. .. .. .. .. .. .. .. 287
10. Dr. Scholefield and Dr. Hooper .. .. .. .. .. .. .. .. .. .. .. .. .. 287
11. Scott on Cove.. .. .. .. .. .. .. .. .. .. .. .. .. .. .. .. .. .. 288
12. Further Testimony of the Remedial Value of the Carbonate of Soda .. .. .. 288

### Extra-Limites.

1. An Account of an Epidemic Cholera which prevailed in the District of Berehaven in Ireland, in the Autumn of 1837. By Edmund Sharkey, M.B. .. .. .. 289

HASLAR HOSPITAL.

2. A Case of Perfect Anchylosis of the Five Superior Cervical Vertebræ to each other, &c. By Stephen S. Stanley, Esq. .. .. .. .. .. .. .. .. .. .. .. .. 296

ADDENBROOKE'S HOSPITAL.

3. Clinical Contributions from Addenbrooke's Hospital, Cambridge, for the Years 1836-37. By H. T. Bond, M.D. .. .. .. .. .. .. .. .. .. .. .. .. .. 299
1. Diseases of the Stomach .. .. .. .. .. .. .. .. .. .. .. .. .. .. 299
A. Functional Derangement of the Stomach .. .. .. .. .. .. .. .. .. 299
B. Inflammatory and Organic Diseases of the Stomach .. .. .. .. .. .. 316
2. Disorders of the Intestines.. .. .. .. .. .. .. .. .. .. .. .. .. .. 323
3. Diseases of the Liver .. .. .. .. .. .. .. .. .. .. .. .. .. .. .. 332
A. Sanguineous Congestion, or Inflammation of the Liver .. .. .. .. .. 333
B. Enlargement or other Organic Disease of the Liver .. .. .. .. .. .. 333
4. On the Best Means of Applying Pressure to the Uterus after Delivery .. .. .. 338
5. "A Crust for the Critics." .. .. .. .. .. .. .. .. .. .. .. .. .. .. .. 341
Bibliographical Record .. .. .. .. .. .. .. .. .. .. .. .. .. .. .. .. 346

